# Indocyanine Green Assisted Removal of Orbital Lacrimal Duct Cysts in Children

**DOI:** 10.1155/2015/130215

**Published:** 2015-01-08

**Authors:** Shay Keren, Gad Dotan, Leah Leibovitch, Dinesh Selva, Igal Leibovitch

**Affiliations:** ^1^Oculoplastic and Orbital Institute and Department of Ophthalmology, Tel Aviv Medical Center, 64239 Tel Aviv, Israel; ^2^The Sackler Faculty of Medicine, Tel Aviv University, Tel Aviv, Israel; ^3^Department of Neonatology, The Edmond and Lily Safra Children's Hospital, Sheba Medical Center, Ramat Gan, Israel; ^4^South Australian Institute of Ophthalmology, Royal Adelaide Hospital, University of Adelaide, Adelaide, SA, Australia

## Abstract

*Aim*. To report on the use of indocyanine green (ICG) during surgical removal of pediatric orbital lacrimal duct cysts. *Method*. We conducted a retrospective review of our cases of surgical excision of orbital lacrimal duct cysts using intraoperative injection of indocyanine green (ICG), which was used following inadvertent cyst rupture and volume loss. The dye allowed complete cyst visualization and complete excision despite volume loss or cyst rupture. *Results*. The study included 6 children (3 boys, mean age of 4.2 ± 0.84 years, range 3–5 years). Mean follow-up period was 9.3 months. All cysts were located in the inferonasal quadrant of the orbit (4 in the right side). In all cases, ICG was injected into the cyst at the time of surgery following unintentional cyst rupture. After the dye injection, it was easy to identify the borders of the cyst, permitting complete cyst removal, without any intra- or postoperative complications. Pathological examination revealed that all cysts were of lacrimal duct origin. *Conclusion*. Intraoperative injection of ICG into orbital cysts in children can aid surgeons in identifying cyst borders following inadvertent rupture, allowing complete removal.

## 1. Introduction

Dermoid cysts, mucocele, and congenital ocular anomalies are the most common orbital cystic lesions in children [1]. However, cysts arising from the lacrimal duct are less frequently encountered [2–5]. These cystic lesions may be congenital, inflammatory, or traumatic in origin. Lacrimal duct cysts can retain communication with the lacrimal system or be completely separated from it. The most common presentation is that of a cystic mass located in the inferior medial orbit, occasionally associated with signs and symptoms of secondary nasolacrimal duct obstruction [6]. Most lesions are small in size and asymptomatic soon after birth; however with time they may enlarge considerably, potentially causing globe compression which requires early resection.

In order to prevent local recurrence, it is necessary to remove the entire cyst at the time of surgery without leaving any residual cyst tissue. On occasions, this may be challenging since cyst boundaries are not easily apparent, especially posteriorly. Inadvertent puncture of the cyst capsule may further limit complete excision.

Indocyanine green (ICG-Akorn Inc., Lake Forest, IL 66045, USA) is a coloring dye that has numerous uses in ophthalmology with established intraocular, topical, and intravenous safety [7]. Reports on the use of ICG during resection of conjunctival cysts were published; however, to the best of our knowledge, there is only one case report on the use of this dye during removal of orbital lesions [8].

The purpose of the study is to report on our experience in using ICG during removal of pediatric orbital cysts, raising the awareness of surgeons for the possibility of its use during such surgery.

## 2. Methods

We reviewed all cases of pediatric orbital lacrimal cysts that were operated using intralesional ICG injection at the time of surgery. Data collection included demographics, clinical history, localization of the lesion, imaging findings, surgical technique, and complications. Pathological analysis, surgical outcome, and duration of follow-up were also recorded. The study was approved by the IRB committee of our medical center and its compliance was in agreement with rules and regulations.

All procedures were performed under general anesthesia. Surgical approach was via a skin incision or through the conjunctiva in the fornix. After the cystic lesion was identified, if leakage was recognized along with loss of cyst volume, ICG dye was injected into the lesion with a 25-gauge needle. Cyst borders were readily apparent even though there was immediate loss of volume subsequent to the injection. Following delineation of the cyst capsule with the green dye, complete excision was performed. The skin or conjunctiva was closed using 7/0 vicryl sutures and the surgical site was bandaged with a topical antibiotic ointment. All children were seen one week after surgery and final assessment of surgical outcome was made at least 6 months afterwards.

## 3. Results

Six children (3 boys) were included in this study. All children had orbital imaging preoperatively; two children had computerized tomography (CT), one had magnetic resonance imaging (MRI), and 3 children had orbital ultrasound. All lesions were unilateral (4 in the right orbit; 2 in the left orbit), always located in the inferomedial orbital quadrant. Four cysts were diagnosed soon after birth due to the presence of medial subcutaneous swelling seen at the area of the lacrimal system, accompanied by cystic sensation upon palpation; however all surgeries were performed after the age of 3 years (mean age 4.2 years ± 0.84, range 3–5 years) when there was evidence of progressive enlargement causing globe compression or cosmetic disfigurement unacceptable to the parents. None of the children were operated on for relief of nasolacrimal duct obstruction.

Surgical excision was performed through the skin in two cases and through the conjunctiva in the remaining four cases. No complications occurred at the time of local ICG injection. ICG was injected when intraoperative loss of cyst volume was noted. None of the cysts were connected to the nasolacrimal duct, and all showed patency of the lacrimal system on syringing after cyst removal, with no evidence of any leakage to the surgical site. Therefore, no silicone tube intubation was required.

A successful outcome was achieved in all cases. In all cases the pathology report confirmed complete excision of a cystic lesion lined with epithelium of lacrimal duct origin. None of the children had cyst recurrence during follow-up (mean follow-up 9 months) or any evidence of nasolacrimal duct obstruction.

### 3.1. Case Presentations

#### 3.1.1. Case  1

**Figure 1:**
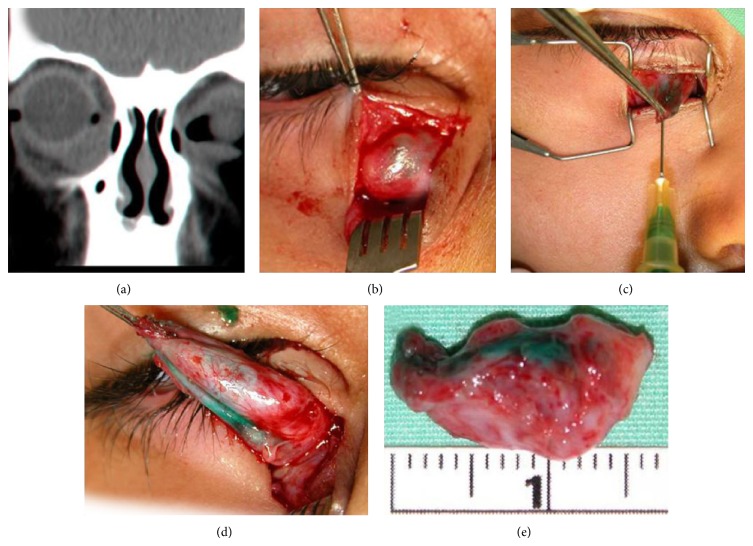
(a) Coronal CT scan showing a 2 cm homogenous inferomedial cystic mass in the right orbit. (b) Cyst exposure through a skin incision. (c) Intralesional injection of ICG. (d) Capsule demonstration and further exposure. (e) The completely excised cyst.

One of the patients had a right inferomedial cystic lesion noted soon after birth. The patient was followed up regularly and when the lesion was growing considerably, an orbital CT was performed demonstrating a 2 cm hyperdense homogeneous mass in the inferomedial aspect of the right orbit, with mild compression of the right eye (Figure 1(a)). The patient had no symptoms or signs of nasolacrimal duct obstruction. At the time of surgery a horizontal skin incision over the cyst was made and the cyst was exposed (Figure 1(b)). Upon further exposure, the cyst started to lose volume, and therefore ICG was injected into the cyst, which allowed improved visualization of the lesion boundaries (Figures 1(c) and 1(d)) permitting its complete excision (Figure 1(e)). The patient was treated twice a day with Maxitrol ointment (dexamethasone with polymyxin B and neomycin) over the surgical wound. Pathology examination identified complete excision of a cyst lined with epithelium of lacrimal duct origin. Postoperatively, the patient was asymptomatic and no cyst recurrence was noted.

#### 3.1.2. Case  2

**Figure 2:**
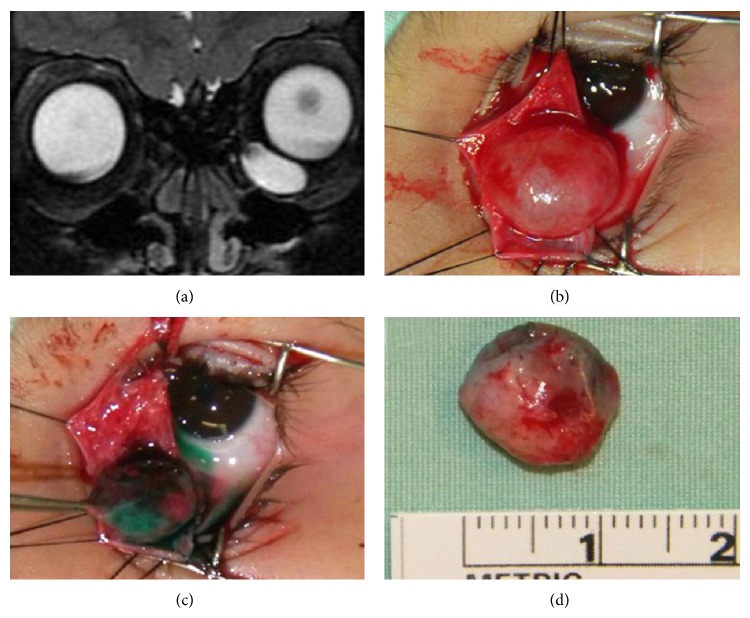
(a) T2 Coronal MRI scan showing a 1.5 cm inferomedial cystic mass in the left orbit. (b) Cyst exposure through a conjunctival incision. (c) Capsule demonstration after intralesional ICG injection. (d) The completely excised cyst.

A second patient was examined in the clinic for evaluation of a left inferomedial orbital cyst that was recognized since the age of 3 months. The patient had a Doppler ultrasound examination which identified a cystic lesion with a clear liquid content. During follow-up there was evidence of progressive enlargement of the lesion. Orbital MRI performed shortly before surgery demonstrated a 0.7 × 1.5 cm oval mass with clear fluid contents located in the left inferomedial orbit, causing mild globe compression (Figure 2(a)). This lesion was surgically resected through a conjunctival incision. ICG was injected into the cyst at the time of surgery when loss of volume was noted by the surgeon (Figures 2(b)–2(d)).

The patient was treated with topical chloramphenicol 5% ointment over the surgical wound. Pathological analysis identified a cyst lined with epithelium of lacrimal duct origin. During the 6-month follow-up period no surgical complications or cyst recurrence were noted.

## 4. Discussion

By using intralesional ICG injection, we were able to completely remove lacrimal duct orbital cysts in children, even though unintentional volume loss occurred earlier, without any complications or recurrence. This dye is frequently used in ophthalmology, including during orbital surgery, with an established safety profile in humans [7].

Kobayashi et al. used ICG during removal of an acquired conjunctival cyst [9]. They noted that following intralesional injection the cyst became clearly visible, and staining remained even after an inadvertent perforation of the cyst capsule that caused immediate collapse. Chan et al. also reported on the use of ICG during similar conjunctival surgery [10]. Others reported on the use of different substances, including sodium hyaluronate or Healon V and Trypan blue during excision of conjunctival cysts [11, 12]. Injecting a mixture of ICG with a viscoelastic material is another useful technique [8]. However, according to our experience there is no need to use a viscoelastic material as well, since it may not be readily available for all orbital surgeons and it increases the cost of surgery.

To the best of our knowledge, our study is the first to report on the use of ICG for demonstration of orbital cysts during pediatric orbital surgery, although its use has been reported earlier in an adult [8]. Using this dye, we were able to completely remove the cysts even though partial or complete collapse of the lesion typically occurred.

In conclusion, we believe that ICG has a role in pediatric orbital lacrimal cyst surgery, helping with improved visualization of the cyst capsule and allowing complete cyst removal, especially with unintentional rupture of the cyst and volume loss that occurred earlier.
